# Genome‐wide mosaicism in divergence between zoonotic malaria parasite subpopulations with separate sympatric transmission cycles

**DOI:** 10.1111/mec.14477

**Published:** 2018-02-13

**Authors:** Paul C. S. Divis, Craig W. Duffy, Khamisah A. Kadir, Balbir Singh, David J. Conway

**Affiliations:** ^1^ Faculty of Medicine and Health Sciences Malaria Research Centre Universiti Malaysia Sarawak Kota Samarahan Malaysia; ^2^ Pathogen Molecular Biology Department London School of Hygiene and Tropical Medicine London UK

**Keywords:** adaptation, genomic divergence, host‐specificity, introgression

## Abstract

*Plasmodium knowlesi* is a significant cause of human malaria transmitted as a zoonosis from macaque reservoir hosts in South‐East Asia. Microsatellite genotyping has indicated that human infections in Malaysian Borneo are an admixture of two highly divergent sympatric parasite subpopulations that are, respectively, associated with long‐tailed macaques (Cluster 1) and pig‐tailed macaques (Cluster 2). Whole‐genome sequences of clinical isolates subsequently confirmed the separate clusters, although fewer of the less common Cluster 2 type were sequenced. Here, to analyse population structure and genomic divergence in subpopulation samples of comparable depth, genome sequences were generated from 21 new clinical infections identified as Cluster 2 by microsatellite analysis, yielding a cumulative sample size for this subpopulation similar to that for Cluster 1. Profound heterogeneity in the level of intercluster divergence was distributed across the genome, with long contiguous chromosomal blocks having high or low divergence. Different mitochondrial genome clades were associated with the two major subpopulations, but limited exchange of haplotypes from one to the other was evident, as was also the case for the maternally inherited apicoplast genome. These findings indicate deep divergence of the two sympatric *P. knowlesi* subpopulations, with introgression likely to have occurred recently. There is no evidence yet of specific adaptation at any introgressed locus, but the recombinant mosaic types offer enhanced diversity on which selection may operate in a currently changing landscape and human environment. Loci responsible for maintaining genetic isolation of the sympatric subpopulations need to be identified in the chromosomal regions showing fixed differences.

## 1 INTRODUCTION

The zoonotic malaria parasite *Plasmodium knowlesi* is a significant cause of human malaria in South‐East Asia. Although long known as a malaria parasite of long‐tailed and pig‐tailed macaques that could potentially infect humans (Coatney, Collin, Warren, & Contacos, 1971), the first large focus of human cases was only detected approximately 15 years ago in Malaysian Borneo (Singh et al., 2004). Since then, infections have been described from throughout Malaysia (Cox‐Singh et al., 2008; William et al., 2013; Yusof et al., 2014) and in almost all countries in South‐East Asia (Singh & Daneshvar, 2013). Indeed, *P. knowlesi* is now the most common cause of human malaria in Malaysia (Barber, Rajahram, Grigg, William, & Anstey, 2017), with infections capable of reaching very high parasitaemia and sometimes leading to the death of patients (Cox‐Singh et al., 2008; Daneshvar et al., 2009; Rajahram et al., 2016; Singh & Daneshvar, 2013; William et al., 2011).

Multilocus microsatellite genotyping analysis of *P. knowlesi* infections revealed that human infections in Malaysian Borneo comprise two major genetic subpopulations that are, respectively, associated with long‐tailed and pig‐tailed macaque reservoir hosts (Divis et al., 2015), with significant divergence confirmed by whole‐genome sequence analyses of parasites in human infections (Assefa et al., 2015). In most areas of Malaysian Borneo, the number of human clinical infections of the parasite subpopulation type associated with long‐tailed macaques (Cluster 1) is higher than those having the type associated with pig‐tailed macaques (Cluster 2) (Divis et al., 2017). Further analyses of additional samples have subsequently revealed a third divergent subpopulation of *P. knowlesi* (Cluster 3) on the mainland of South‐East Asia which includes Peninsular Malaysia (Divis et al., 2017; Yusof et al., 2016). So far, only *P. knowlesi* parasites of Cluster 3 have been studied in infections of laboratory monkeys (Assefa et al., 2015), and one strain of this type has been adapted to efficiently invade human erythrocytes in culture (Lim et al., 2013; Moon et al., 2013). To develop laboratory studies on the other two major zoonotic populations will require establishment of parasite isolates in controlled monkey infections, or ideally into culture with erythrocytes. Analysis of *P. knowlesi* samples from human clinical infections is relatively straightforward, as most of these are not mixed with other species, whereas most natural *P. knowlesi* infections in macaques occur together with other primate malaria parasite species (Lee et al., 2011).

The first large‐scale whole‐genome sequence analysis of *P. knowlesi* infections contained clinical samples that were mostly of the Cluster 1 type (*N* = 38), yielding results indicating that this has undergone long‐term population growth, with additional evidence of selection on particular loci (Assefa et al., 2015). There were only 10 Cluster 2 type infections sequenced in the study, which limited investigation of the demographic history of that subpopulation, but these were sufficient to indicate that the level of intercluster divergence varied across the genome, some loci having a concentration of apparently fixed differences and others showing more shared polymorphism (Assefa et al., 2015). A separate simultaneous study reported data from another six infections, confirming the divergence between sympatric subpopulations (Pinheiro et al., 2015), but this did not cumulatively give a much deeper sample. In agreement with the initial study (Assefa et al., 2015), a recent secondary analysis of the previously published data confirmed the existence of genomic regions with shared polymorphisms (Diez Benavente et al., 2017), but did not include any new data.

For a more informed comparison of these important zoonotic parasite subpopulations, a much larger sample of Cluster 2 type *P. knowlesi* genome sequences was obtained in this study. Combining the new data with samples sequenced previously (Assefa et al., 2015; Pinheiro et al., 2015) yielded a total of 34 Cluster 2 genome sequences that enables a more comprehensive analysis of genomic polymorphism and divergence between the subpopulations. This provides new understanding of the genome‐wide variation in divergence of these two sympatric *P. knowlesi* subpopulations, essential for understanding their long‐term maintenance and potential for future adaptation.

## 2 MATERIALS AND METHODS

### 2.1 New *P. knowlesi* DNA samples selected for analysis

Venous blood samples were obtained from patients infected with *P. knowlesi* malaria at Kapit Hospital in Sarawak between March and November 2014, after written informed consent from each patient had been obtained. The collection of blood samples was approved by the Medical Research and Ethics Committee of the Malaysian Ministry of Health and by the Ethics Committee of the London School of Hygiene and Tropical Medicine. Leucocytes were removed by allowing 10 ml of blood to pass through a CF11 cellulose column, to enrich for erythrocytes and thereby increase the proportion of parasite compared to host DNA. Genomic DNA was extracted using QIAamp DNA Mini kits (Qiagen, Germany), and all infections were confirmed to contain only *P. knowlesi* by nested PCR assays testing for all locally known malaria parasite species (Lee et al., 2011). Determination of the genetic subpopulation cluster of each DNA sample was conducted by microsatellite genotyping (Divis et al., 2017), and 21 samples of the Cluster 2 type that had sufficient DNA were selected for whole‐genome sequencing. These were mostly single genotype infections as determined by microsatellite typing (Divis et al., 2017).

### 2.2 *P. knowlesi* whole‐genome sequencing

DNA libraries were constructed using the TruSeq Nano DNA Library Preparation Kit (Illumina, San Diego, CA, USA). Physical shearing of the genomic DNA into fragments having an average size of 550 bp was performed using a M220 Focused‐ultrasonicator (Covaris, USA). After denaturation at 95°C for 3 min, amplification of genomic DNA was performed with low number of PCR cycles (eight cycles at 98°C for 20 s, 60°C for 15 s and 72°C for 30 s) followed by a 72°C completion for 5 min. The quality of DNA libraries was assessed using the Agilent High Sensitivity DNA kit (Agilent Technologies, Santa Clara, CA USA), while quantitation was performed using the KAPA Library Quantification Kit for Illumina^®^ platform (KAPA Biosystems, Boston, MA, USA). All libraries were then normalized to 4 nm, and up to 12 samples were included on each sequencing run. Paired‐end whole‐genome sequencing was performed on pooled DNA libraries using MiSeq Chemistry version 3 reagents, on the MiSeq platform (Illumina, San Diego, CA, USA) with a read length of 300 bp. Raw data of short reads generated in FASTQ format were undergone for quality check using the trimmomatic software (Bolger, Lohse, & Usadel, 2014) with defined parameters (LEADING:3 TRAILING:3, SLIDINGWINDOW:4:10 MINLEN:36).

Trimmed FASTQ reads for individual isolates were then aligned against the version 2.0 of *P. knowlesi* strain H reference genome (http://www.genedb.org/Homepage/Pknowlesi, genome annotation March 2014, accessed December 2015) using the burrows‐wheeler aligner software version 0.7 with the BWA‐MEM algorithm and default parameters (Li, 2013). This generated file in the SAM (sequence alignment/map) format, and followed by the conversion into a BAM (binary alignment/map) format using the samtools package version 0.1 (Li et al., 2009). Due to the possible effect of PCR amplification bias introduced during the DNA library preparations, read duplications were removed using the “*MarkDuplicates”* command from the Picard toolkit (https://github.com/broadinstitute/picard). The average depth coverage was analysed by the bedtools version 2 package using the “genomeCoverageBed” command (Quinlan & Hall, 2010).

Re‐mapping of short read genome sequences generated from previous studies (Assefa et al., 2015; Pinheiro et al., 2015) against the version 2.0 of *P. knowlesi* strain H reference genome was also performed in the analysis (Table S1). These include 48 isolates from Kapit and Betong in Malaysian Borneo (Sequence Read Archive numbers ERR985372–ERR985419) representing Cluster 1 and Cluster 2 type parasites collected between 2008 and 2013, six isolates from Sarikei in Malaysian Borneo (SRA numbers ERR274221, ERR274222, ERR274224, ERR272225, ERR366425 and ERR366426) and five laboratory isolates (“Nuri” SRA numbers ERR019406, “Hackeri” SRR2221468, “Malayan” SRR2225467, “MR4‐H” SRR2225571 and “Philippines” SRR2225573). The reference H strain sequence belongs to Cluster 3 (Assefa et al., 2015), which is approximately equally divergent from Clusters 1 and 2, so no bias is expected in the efficiency of mapping of the sequences to this reference.

### 2.3 Single nucleotide polymorphism calling and filtration

The calling of high‐quality single nucleotide polymorphisms (SNPs) was performed using several steps, following procedures described previously (Assefa et al., 2015). For each isolate, SNPs were first identified from the BAM file using samtools/bcftools with the following parameters: *mpileup –B –Q 23 –d 2000 –C 50 –ugf; varFilter –d 10 ‐D 2000*. A high‐quality list of potential variant positions (Phred quality, *Q* > 30) was extracted from the resulting variant call format (VCF) file, and a list of unique SNP lists was generated by concatenating all variant positions from all isolates. Using these unique SNP positions, the mapping quality (mq) and base quality (bq) were checked for each isolate to remove positions with an excess of low‐quality reads with the requirement of the minimum read depth coverage at 10x. The ratio of read depth values at high‐quality (mq = 26; bq = 23) and low‐quality (mq = 0; bq = 0) thresholds were calculated for each isolate using customized Perl scripts, and any SNP positions with the ratio below 0.5 were discarded.

Further filtration involved the removal of positions that contained ambiguous sequences (represented as a long stretch of unknown nucleotides “N”) in the reference genome. The *SICAVar*,* KIR*, and *pk‐fam‐a* to *pk‐fam‐e* multigene families (Pain et al., 2008) and the subtelomeric regions were also filtered out to avoid ambiguous alignments, which may cause false‐positive SNP calls. Subtelomeric regions were here determined by visually inspecting the whole‐genome synteny mapping of *P. knowlesi* with the *P. vivax* homolog using the PlasmoDB GBrowse v2.48 (plasmodb.org/cg‐bin/gbrowse/plasmodb/), with the boundaries of subtelomeric regions defined as sequences adjacent to the first conserved protein‐coding gene (Table S2). After exclusion of subtelomeric regions and the large multigene families, 21.2 Mb (92%) of the 23.0 Mb corresponding to the reference nuclear genome was analysed from each sample.

### 2.4 Genomic diversity and population structure

To measure the amount of polymorphism within the parasite population, the average pairwise nucleotide diversity (π) among the sequences from the individual infection samples was calculated. The skewness in allele frequency distributions was estimated by Tajima's *D* index. Both indices were calculated using the same genome‐wide SNP data set in nonoverlapping window sizes of 10 kb and performed using the divstat software (Soares, Moleirinho, Oliveira, & Amorim, 2015). To illustrate the population substructure, the matrix of pairwise DNA distance among individuals was calculated and the Neighbour‐Joining tree was constructed using the ape package version 3.4 in the R environment (Paradis, Claude, & Strimmer, 2004). An independent population structure evaluation was also conducted using principal coordinate analysis (PCoA) with SNPs having no missing data, using the ape package.

To estimate the divergence between the subpopulations, the genome‐wide distribution of the fixation index (*F*_*ST*_) between the two‐subpopulation clusters was computed with SNPs having minor allele frequencies (MAFs) above 0.1, and above 0.3, using customized R functions. An elevated *F*_*ST*_ threshold was set at the 90th percentile of the *F*_*ST*_ distributions for all SNPs. Average *F*_*ST*_ values were calculated in windows of 500 SNPs with sliding by 250 SNPs. The *F*_*ST*_ values for each window were tested for high‐ or low‐differentiated regions against the genome‐wide mean *F*_*ST*_ value.

Genomic regions with contrasting levels of intercluster divergence were determined empirically by examining the *F*_*ST*_ distribution across the genome at two different MAFs (MAF above 0.1 and 0.3). For each MAF analysis, average *F*_*ST*_ values were calculated in windows of 200 SNPs (sliding by 100 SNPs), 500 SNPs (sliding by 250 SNPs) and 1,000 SNPs (sliding by 500 SNPs). Mean global *F*_*ST*_ values and window *F*_*ST*_ values were then converted into standard *z*‐scores in order to standardize the definition of outlier windows for different parameters. Regions of high‐ or low‐*F*_*ST*_ windows were observed and compared among the analyses that used different MAF parameters.

Genomic regions were categorized into low divergence regions (LDR; *z*‐scores < −0.5), intermediate divergence regions (IDR), and high divergence regions (HDR; *z*‐scores > 0.5). To determine the contiguous extent of these regions in detail, adjacent outlier windows were merged to form larger adjoining regions. Peak and trough patterns of window *z*‐scores around the thresholds (*z*‐scores < −0.5 and *z*‐scores > 0.5) were taken into consideration in determining the junctions. Each candidate region was demarcated by first and last SNPs that fell within the merged windows, except for HDRs where SNPs with elevated *F*_*ST*_ values were used as start and endpoints.

Patterns of polymorphisms (nucleotide diversity summarized by π and allele frequency spectrum summarized by Tajima's *D*) in all genomic regions were evaluated using divstat software. Test runs were performed in nonoverlapping window sizes of 10 kb for each subpopulation. Nonparametric Kruskal–Wallis tests were used to compare among the genomic regions as well as against the genome‐wide background.

### 2.5 Extra‐chromosomal genomes

Population structure and relationships of the sympatric *P. knowlesi* subpopulations were further analysed using the extranuclear DNA, consisting of the nonrecombining genomes of mitochondria and plastid‐like apicoplast. The 5.9‐kb mitochondrial DNA sequences were obtained from the present whole‐genome sequence data and previously published sequences (Assefa et al., 2015; Jongwutiwes et al., 2005; Lee et al., 2011; Pinheiro et al., 2015). Complete mitochondrial sequences were obtained from GenBank database, consisting of 26 haplotypes from human isolates (Accession nos. EU880446–EU880470) and 20 haplotypes from macaque isolates (EU880471–EU880474, EU880477–EU880486, EU880489–EU880493 and EU880499) in Kapit of Malaysian Borneo, and one human isolate from Thailand (AY598141). Three species, *P. coatneyi* (AB354575), *P. cynomolgi* (AB434919) and *P. vivax* (AY791551), that have close evolutionary relationships with *P. knowlesi* were included in the analysis as out‐groups. For the apicoplast genome of *P. knowlesi*, 30.6 kb of the DNA sequences that had clear alignment was extracted from the present whole‐genome data set as well as from previous data (Assefa et al., 2015; Pinheiro et al., 2015) following mapping and base quality checks as mentioned above.

The derived mitochondrial and apicoplast genome sequences were separately aligned using the clustalx programme version 2 (Larkin et al., 2007), following which nucleotide diversity (π) and haplotype diversity (*Hd*) was determined using the dnasp version 5 software (Librado & Rozas, 2009). A maximum‐likelihood tree was inferred with 1,000 bootstrap replicates and gaps treated as missing data using the phangorn packages in R (Schliep, 2011), with the ModelTest algorithm used to determine the best‐fit nucleotide substitution model, which was GTR+I+G (General Time Reversible model with a proportion of invariable sites and gamma distribution). For the mitochondrial sequences, major haplotypes were determined with gaps treated as missing data, and the statistical parsimony haplotype network was constructed using the tcs version 1.21 software (Clement, Posada, & Crandall, 2000).

## 3 RESULTS

### 3.1 Generation of new whole‐genome sequences and SNP genotyping

Paired‐end Illumina sequencing of 21 new *P. knowlesi* clinical infection samples, selected on the basis of microsatellite genotyping as belonging to Cluster 2 (the type previously associated with pig‐tailed macaque as well as human infections), yielded a mean of 6.95 million high‐quality reads per sample, which were mapped against the *P. knowlesi* H strain version 2.0 reference genome sequence (Table S3). The mean depth of sequence coverage genome‐wide was 52.3‐fold (range from 28.7‐ to 80.3‐fold) per sample. In addition, Illumina short read sequence data from another 59 *P. knowlesi* isolates obtained previously (Assefa et al., 2015; Pinheiro et al., 2015) were remapped against the *P. knowlesi* H strain version 2.0 reference genome using the same assembly parameters (Table S1), followed by SNP calling. In the combined data set of 80 infection sequences, a total of 2,109,937 SNPs were identified in the nuclear genome. Following exclusion of those in subtelomeric regions or in the *KIR* or *SICAVAR* multigene families, or that had more than two alleles, 1,669,533 SNPs remained, of which 1,186,073 high‐quality SNPs with less than 10% missing calls in all isolates were used for population genomic analyses.

### 3.2 Population genetic structure

**Figure 1:**
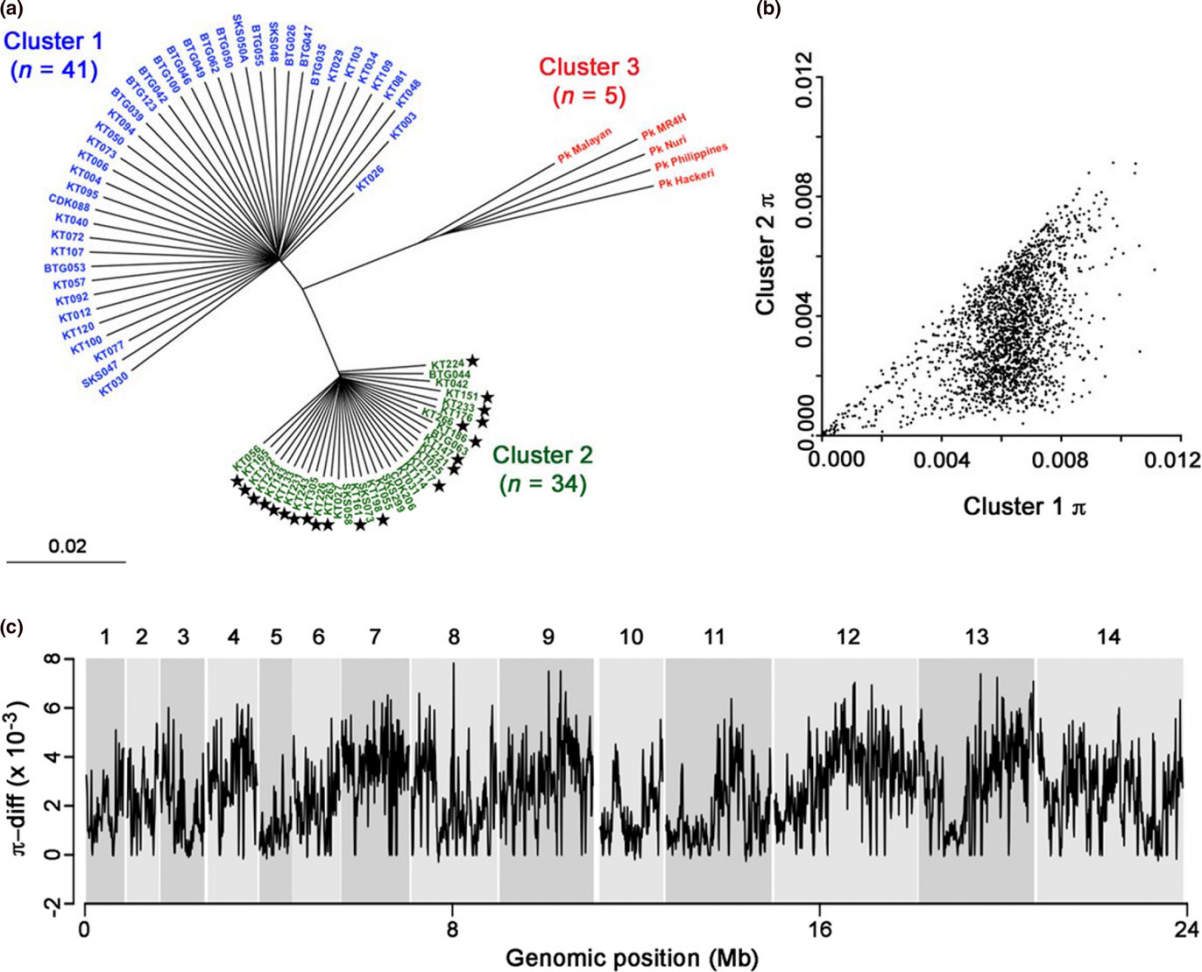
Population structure of *Plasmodium knowlesi* indicated by whole‐genome sequence data. (a) Neighbour‐Joining tree based on a pairwise single nucleotide polymorphism (SNP) difference matrix of 80 *P. knowlesi* isolates. The 21 new genome sequences are indicated with stars, yielding a total sample size for Cluster 2 (*N* = 34) that is similar to that of Cluster 1 (*N* = 41). The scale bar indicates proportions of all SNPs differing between samples. (b) Scatterplot of nucleotide diversity (π) in individual nonoverlapping 10‐kb windows of the genome, comparing data for Cluster 1 and Cluster 2 subpopulations. (c) Differences in nucleotide diversity between Cluster 1 and Cluster 2 subpopulations (π‐diff) in each of the 10‐kb windows of the genome [Colour figure can be viewed at http://wileyonlinelibrary.com]

Consistent with predictions from cluster assignment based on microsatellite genotyping, all 21 of the new *P. knowlesi* clinical infection samples showed genome sequences belonging to the Cluster 2 subpopulation (Figures 1a and S1). Together with previous data, this yielded an overall sample of 34 Cluster 2 isolate sequences, to achieve a similar sample size as previously available for Cluster 1. As is visually apparent from the Neighbour‐Joining tree based on the pairwise genetic distances (Figure 1a), the Cluster 2 infections are less genetically diverse (π = 3.43 × 10^−3^) than the Cluster 1 infections (π = 5.78 × 10^−3^). Furthermore, the Cluster 1 subpopulation demonstrated a homogenous pattern of sequence diversity across the 14 chromosomes (Kruskal–Wallis, *p* = .23), in contrast with Cluster 2 that showed heterogeneous levels of diversity across the chromosomes (Kruskal–Wallis *p* < 10^−16^) (Figure S2). In Cluster 2, nucleotide diversity of entire chromosomes ranged from 2.25 × 10^−3^ (for chromosome 7) to 4.38 × 10^−3^ (for chromosome 5), but all had a lower diversity than in Cluster 1 (Wilcoxon signed rank *p* < 10^−16^). In a majority of nonoverlapping 10‐kb windows genome‐wide, nucleotide diversity (π) indices were lower in Cluster 2 (Figure 1b). Large regions of chromosomes showed contiguous stretches in which diversity was much higher in Cluster 1, and also contiguous stretches in which the diversity was more similar (Figure 1c).

### 3.3 Genomic regions of high and low divergence

**Figure 2:**
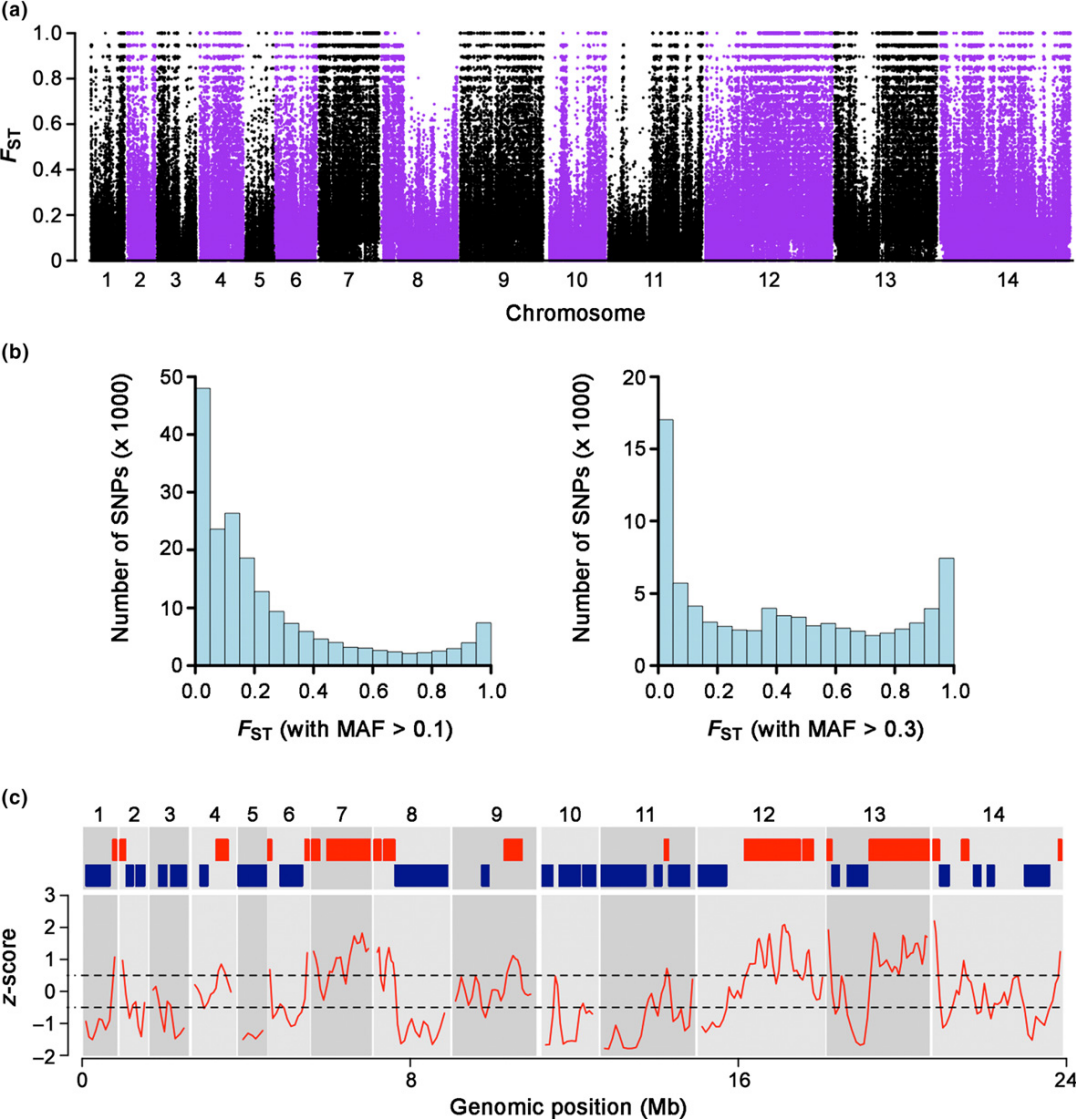
Genome‐wide plot of divergence between the sympatric *Plasmodium knowlesi* Cluster 1 and Cluster 2 subpopulations in Malaysian Borneo. Each dot shows the *F*_*ST*_ value of an individual single nucleotide polymorphism (SNP), of 193,068 SNPs with minor allele frequencies above 0.1. The overall genome‐wide mean *F*_*ST*_ value is 0.25. (b) Strong bimodal frequency distribution of *F*_*ST*_ values for SNPs genome‐wide. The left plot shows the distribution of values for 193,068 SNPs with minor allele frequencies (MAF) > 0.1, and the right plot shows the distribution of values for 80,168 SNPs with MAF > 0.3 (the genome‐wide average *F*_*ST*_ value was 0.42 for SNPs with MAF > 0.3). (c) Contiguous regions of high and low divergence throughout the genome identified by analysis of *F*_*ST*_ values of windows of 500 consecutive SNPs converted to standardized z‐scores. Thresholds of 0.5 standard deviations above and below the genome‐wide average *F*_*ST*_ demarcate the regions of high divergence (red blocks) and low divergence (dark blue blocks) [Colour figure can be viewed at http://wileyonlinelibrary.com]

The genome‐wide variation in diversity in Cluster 2 suggested that there might be variation in levels of intercluster divergence. Analysing SNPs with overall minor allele frequencies above 10% (193,068 SNPs), the mean genome‐wide fixation index indicated substantial divergence between the two subpopulations (mean *F*_ST_ = 0.25; Figure 2a). The frequency distribution of *F*_*ST*_ values was bimodal, one peak having values just above zero and a second peak having values at or approaching 1.0 (Figure 2b). Very high intercluster *F*_*ST*_ values of > 0.8 were seen for 19,116 SNPs, and 7,415 (3.8%) showed complete fixation of alternative alleles (*F*_ST_ = 1.0). A large proportion of low *F*_*ST*_ values were removed when analysis focused on SNPs with overall allele frequencies of >0.3 (Figure 2b). Mean *F*_*ST*_ values for whole chromosomes ranged from 0.09 (for chromosome 5) to 0.40 for (chromosome 7).

The relative level of population differentiation of all windows of 500 contiguous SNPs across the genome was evaluated by considering standard deviations from the mean genome‐wide *F*_*ST*_ value (*z*‐score). Genomic regions were identified that contained contiguous windows defining low divergence regions (LDR with *z*‐score < −0.5) and high divergence regions (HDR with *z*‐score > 0.5). This revealed large genomic blocks of high or low divergence (Figure 2c; Table S4). For example, chromosomes 7, 12 and 13 had HDRs covering most of their respective lengths, whereas chromosomes 3, 5 and 10 showed no HDRs (Figure 2c).

### 3.4 Intracluster diversity in genomic regions with contrasting levels of divergence

**Figure 3:**
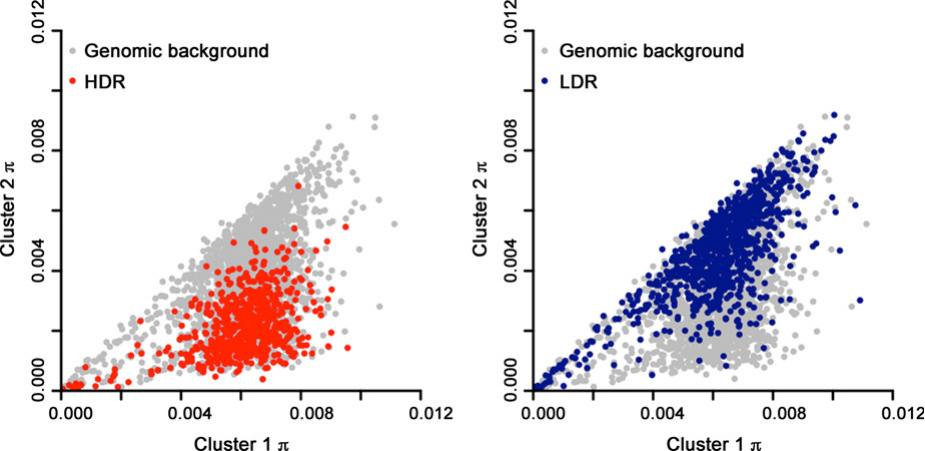
Sequence diversity of *Plasmodium knowlesi* Cluster 2 is lowest in regions of the genome that have highest fixation indices in comparison with Cluster 1. Scatterplots show nucleotide diversity in discrete 10‐kb windows genome‐wide, with red points (left) showing windows in high divergence regions (HDR) and blue points showing windows in low divergence regions (LDR). For HDR, mean π* = *5.80 × 10^−3^ for Cluster 1 and 2.08 × 10^−3^ for Cluster 2. For LDR, mean π* = *5.60 × 10^−3^ for Cluster 1 and 4.14 × 10^−3^ for Cluster 2 [Colour figure can be viewed at http://wileyonlinelibrary.com]

The relationship of intercluster divergence with the varying nucleotide diversity (π) in Cluster 2 across the genome (Figure 1c) was investigated. Comparing between the two subpopulations, the differences in nucleotide diversity were higher in the HDRs than in the LDRs or in the rest of the genome (Figure 3; Mann–Whitney *U p* < 10^−16^ for both comparisons). Most of the highly differentiated regions were those in which nucleotide diversity was substantially lower in Cluster 2 (Figure 3).

Reduced nucleotide diversity in HDRs compared to the rest of the genome was specifically seen in Cluster 2 (mean π in HDRs = 2.08 × 10^−3^; Mann–Whitney *p* < 2.2 × 10^−16^), and not in Cluster 1 (mean π in HDRs = 5.80 × 10^−3^; Mann–Whitney *p* = 0.25). Similarly, higher nucleotide diversity in LDRs compared to the rest of the genome was seen specifically within Cluster 2 (Mann–Whitney *p* = 2.2 × 10^−16^), and not in Cluster 1 (Mann–Whitney *p* = .77).

**Figure 4:**
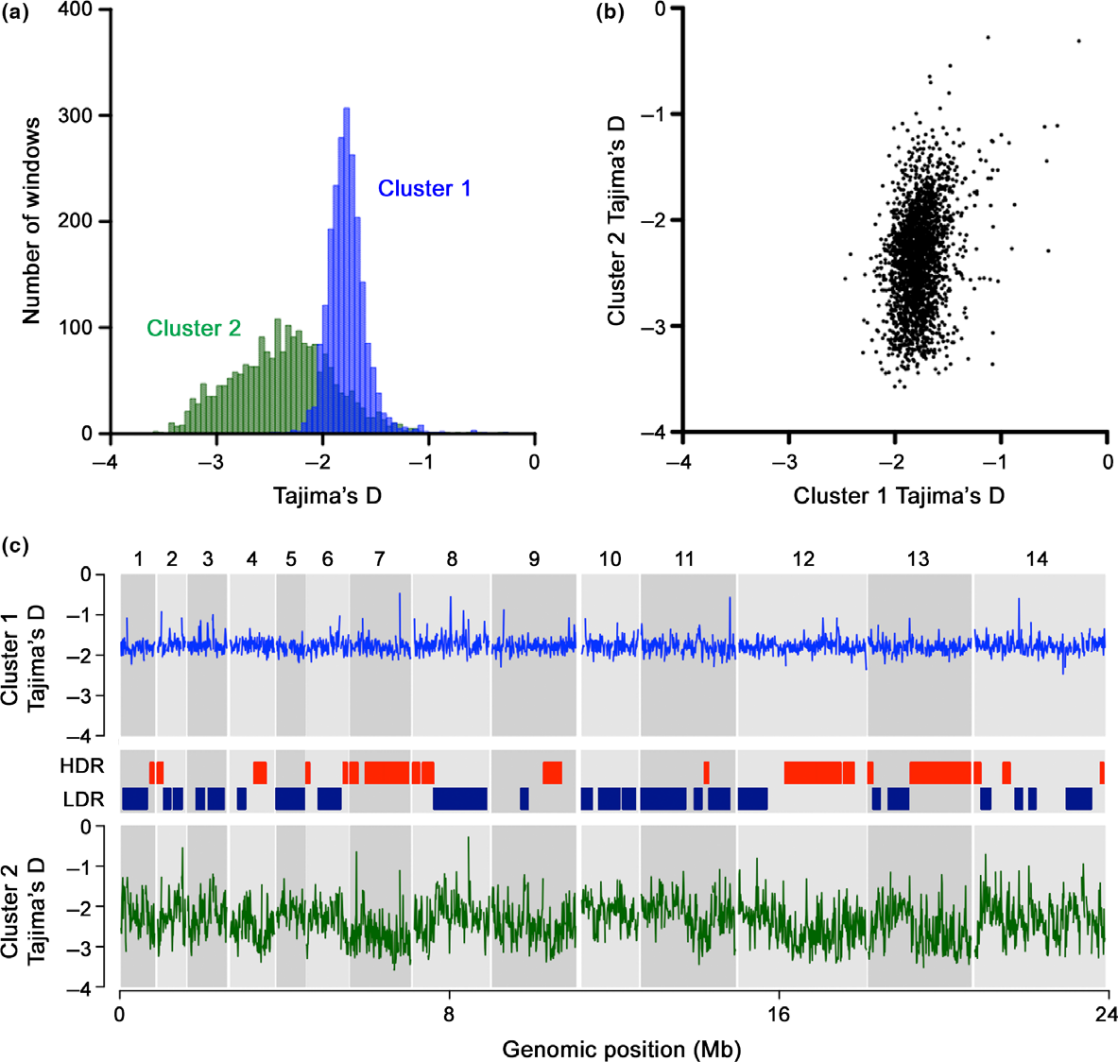
Comparison of genome‐wide Tajima's *D* distributions between the two major *Plasmodium knowlesi* genetic subpopulations in Malaysian Borneo. (a) Frequency distribution of Tajima's *D* values in nonoverlapping 10‐kb windows for Cluster 2 shows more negatively skewed values compared to Cluster 1. (b) Tajima's *D* values for individual 10‐kb windows show a weak correlation between the two subpopulations (Spearman's ρ = 0.25), although this is highly significant (*p* < 10^−16^). (c) Distribution of Tajima's *D* values in nonoverlapping 10‐kb windows across all 14 chromosomes presented in alternate dark and light grey blocks. The mean genome‐wide value for Cluster 1 is −1.77 and for Cluster 2 is −2.37 [Colour figure can be viewed at http://wileyonlinelibrary.com]

Both subpopulations showed strong skew towards low‐frequency variants, with mean Tajima's *D* values of 10‐kb windows of the genome for the Cluster 2 subpopulation being even lower than for the Cluster 1 subpopulation (Figure 4a; Cluster 1 mean *D *=* *−1.77; Cluster 2 mean *D *=* *−2.37; Wilcoxon Signed Rank *p* < 10^−16^). Across all 10‐kb windows in the genome, there was a weak but highly significant correlation in the distribution of Tajima's *D* values in the two clusters (Figure 4b; Spearman's ρ = 0.25; *p* < 10^−16^). The allele frequency spectrum as summarized by Tajima's *D* index was less variable across the 14 chromosomes within the Cluster 1 subpopulation (Kruskal–Wallis *p* = 8.4 × 10^−5^) compared to the Cluster 2 subpopulation (Kruskal–Wallis *p* = 1.6 × 10^−16^) (Figure 4c).

The mosaic pattern of genomic diversity in the Cluster 2 subpopulation suggests that a genome‐wide scan to identify individual genes with exceptionally high values of Tajima's *D* may not be a robust means of identifying genes under balancing selection within this subpopulation, although the approach may be more straightforwardly applied to the Cluster 1 subpopulation (Assefa et al., 2015). However, the *msp1* merozoite surface protein antigen gene that was previously shown to have a high Tajima's *D* value in Cluster 1 also had a high value in the Cluster 2 subpopulation (*D *=* *1.01), suggesting it is likely to be under balancing selection in both. Interestingly, the *ama1* apical membrane antigen gene that did not have a high value in Cluster 1 had an exceptionally high value in Cluster 2 here (*D *=* *1.64). The *csp* circumsporozoite protein gene, that had the highest Tajima's *D* value of all genes in Cluster 1, did not have any detected nonrepeat sequence SNPs in Cluster 2. Thus, although an unbiased comparison cannot be straightforwardly performed, these examples indicate that there are some similarities as well as differences in the strength or targets of balancing selection on antigens in the two different parasite subpopulations.

### 3.5 Phylogeny and introgression of extra‐chromosomal genomes

**Figure 5:**
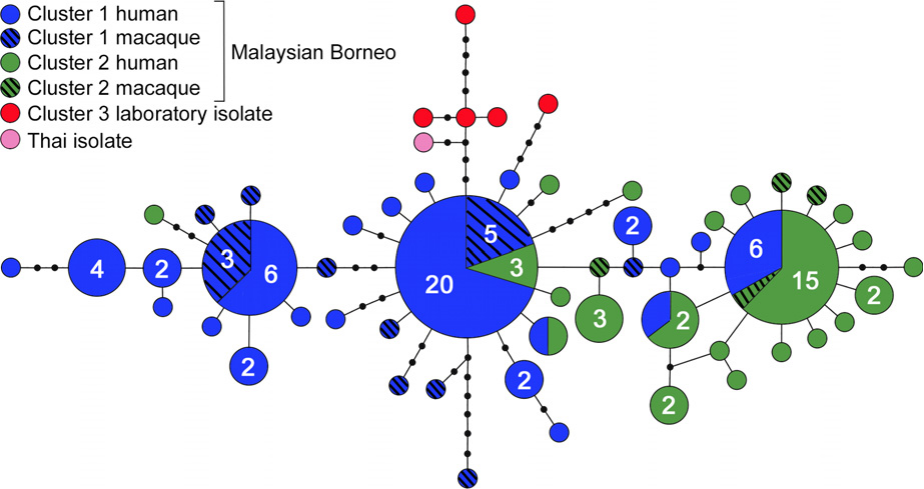
Genealogical network based on 129 *Plasmodium knowlesi* mitochondrial DNA genome sequences showing 56 different haplotypes. Sizes of the circles represent relative numbers of samples with each haplotype, with numbers specified where this is more than one. Connecting lines each represent one mutational step, and black dots represent missing intermediate haplotypes [Colour figure can be viewed at http://wileyonlinelibrary.com]

The analyses of population structure were extended using the maternally inherited extra‐chromosomal genomes. Combination of the 5.9‐kb mitochondrial sequences generated in this study with previously published sequences yielded a sample size of 129 in total and identification of 77 SNPs. These mitochondrial sequences had a global average nucleotide diversity (π) of 7.9 × 10^−4^, with higher values in samples from parasites in Cluster 1 (π = 6.8 × 10^−4^, *n* = 74) than in Cluster 2 (π = 4.9 × 10^−4^, *n* = 46). The genealogical network of mitochondrial genomes contained 56 different haplotypes (Figure 5). The most common and central core haplotype was detected mainly in parasites of the Cluster 1 subpopulation (25 of 28 isolates). A second common haplotype that was more peripheral in the network was seen mostly in the Cluster 2 subpopulation (15 of 21 isolates), while the third common haplotype was distantly related to this and detected only in Cluster 1 (nine isolates). Most of the closely related haplotypes to each of these were also seen only in the corresponding subpopulation clusters, but there is a group of closely related haplotypes internal in the network seen in parasites of Cluster 1 (13 isolates) which is embedded in part of the network that is otherwise only seen in Cluster 2 parasites (Figure 5). Conversely, a few Cluster 2 isolates have haplotypes that are related to those only seen in Cluster 1. A separate branch of haplotypes was seen in laboratory isolates that had mostly been collected from Peninsular Malaysia. Maximum‐likelihood phylogenetic analysis yielded a similar pattern, with haplotype clades being associated but not completely fixed between the Cluster 1 and Cluster 2 subpopulations (Figure S3).

Polymorphism in 30.6 kb of the apicoplast genome could be characterized using the Illumina short read sequence data to identify 520 polymorphic SNPs. With these data, 65 of the 80 isolates were analysed in detail as they had less than 20% missing SNPs, while the remaining 15 samples with more missing SNP data were excluded. The overall nucleotide diversity (π) was 1.79 × 10^−3,^ and this was higher among the Cluster 1 samples (π = 1.77 × 10^−3^) than Cluster 2 samples (π = 1.12 × 10^−3^). Two major lineages were seen, one of which consisted predominantly of Cluster 1 samples, and the other mainly of Cluster 2 samples (Figure S4), although there were several isolates that had haplotypes of the opposite type to that expected for each cluster.

## 4 DISCUSSION

This study analyses the largest ecological sample of sequences representing different subpopulations of a zoonotic eukaryotic parasite species. Whole‐genome sequencing of new samples from one of the major genetic subpopulations of *P. knowlesi* has clearly revealed the genome‐wide patterns of divergence between the sympatric subpopulations, which illuminates aspects of their population history and is essential for understanding their adaptive potential. This provides the most informative overall analysis of population structure of *P. knowlesi* to date, extending the understanding of defined subpopulation clusters that were previously described (Assefa et al., 2015; Divis et al., 2017). These results confirm the distinctness of the two sympatric divergent *P. knowlesi* subpopulations in Malaysian Borneo, supporting the occurrence of independent zoonotic cycles associated with different macaque reservoir host species (Divis et al., 2015; Muehlenbein et al., 2015).

The high differentiation between these two sympatric subpopulations indicates minimal or no ongoing gene flow occurring between them, and a large number of SNPs showed complete fixation of alternative alleles. However, the pattern of divergence was heterogeneous and bimodally distributed, with large regions of exceptionally high or low divergence interspersed throughout the genome. Reduced genetic diversity of the Cluster 2 subpopulation in highly diverged regions suggests there may have been an initial bottleneck in the formation of this subpopulation. The overall allele frequency spectra were negativly skewed for both subpopulations, signifying long‐term population growth, although this was more extreme for the Cluster 2 subpopulation. This gives a more detailed perspective than that previously obtained by analysis of mitochondrial genome sequences, which had already indicated a historical population expansion (Lee et al., 2011). The mitochondrial and apicoplast genomes in *Plasmodium* are inherited together through the female parasite gamete in each transmission cycle (Lim & McFadden, 2010) with negligible recombination at the population level, but analyses of these extra‐chromosomal genomes here indicates some sharing of different haplotypes between the *P. knowlesi* subpopulations. The mosaic pattern with adjacent large regions of alternating high and low diversity in the genome sequences of the Cluster 2 subpopulation, in contrast to the more consistent high diversity throughout the genome for the Cluster 1 subpopulation, suggests that introgression has probably occurred recently from Cluster 1 into the Cluster 2 population.

Despite the differences at the genomic level, it is not yet known whether these two major sympatric subpopulations exhibit significant phenotypic differences, apart from the previously described association with different macaque reservoir host species (Divis et al., 2015, 2017; Lee et al., 2011). Human *P. knowlesi* infections have been associated with a wide spectrum of disease (Cox‐Singh et al., 2010; Daneshvar et al., 2009; Rajahram et al., 2012; William et al., 2011), and there is recent evidence that asymptomatic infections may be more common than previously expected (Fornace et al., 2015; Lubis et al., 2017; Siner et al., 2017), so conducting detailed clinical studies on individuals infected with each parasite subpopulation type is now a priority.

A recent study suggests a link between local deforestation and incidence of *P. knowlesi* infections in an area of Sabah state within Malaysian Borneo (Fornace et al., 2016). Of relevance to this, long‐tailed macaques and pig‐tailed macaques show different habitat ranges in forested and nonforested areas (Moyes et al., 2016), suggesting that there may be micro foci of infection for each subpopulation cluster, and highlighting the need to examine changes over time. It is clear that future research should include monitoring the proportions of the different *P. knowlesi* subpopulations over time, and potential changes in their genetic composition. Sequencing of *P. knowlesi* genomes from natural macaque infections would be more challenging, given that these are usually coinfections together with other primate malaria parasite species (Lee et al., 2011), although new methods of sequencing genomes from single parasites could be adapted to address the issue (Trevino et al., 2017). This would ideally be done alongside sampling of infections in local mosquito vector species that could potentially be maintaining the separate zoonotic transmission cycles.

The genome‐wide mosaicism, showing bimodal levels of divergence as well as limited discordant occurrence of extra‐chromosomal genome lineages, indicate that introgression is likely to have occurred recently between these parasite subpopulations. The recombinant genomes that are now circulating offer a great diversity on which selection may operate, but there is no evidence yet of specific adaptation at introgressed loci. A recent re‐analysis of previously published data identified a common shared haplotype in a chromosomal region with low divergence between the subpopulations (Diez Benavente et al., 2017), although an observation that the region had a slightly higher than background proportion of genes predicted to be expressed at a particular developmental stage may not be relevant, as an extended haplotype may result from selection on a single locus rather than on multiple genes.

In contrast, it is likely that at least one of the chromosomal regions showing fixed differences between the clusters contains a locus responsible for maintaining genetic isolation of the sympatric subpopulations, potentially due to transmission in different mosquito vectors, as well as likely adaptation to the different reservoir macaque hosts. Parasites from these sympatric subpopulations have not yet been studied in laboratory infections or adapted to culture, which will be necessary to define phenotypes and enable experimental analyses of differences between them. Despite major technical challenges of such work, efforts should prove worthwhile, as they are likely to reveal parasite phenotypes not present in the old laboratory lines which were sampled from a different part of the parasite species range (Dankwa et al., 2016; Moon et al., 2016). If there are no parasite subpopulation‐specific barriers to infection of mosquito vectors that may be experimentally used, such as *Anopheles cracens* (Amir, Sum, Lau, Vythilingam, & Fong, 2013), it may ultimately be possible to map loci controlling key phenotypes by performing genetic crosses between parental parasites representing the different subpopulations.

## DATA ACCESSIBILITY

Paired‐end short read genome sequence data for the new parasite infection isolates listed in Table S3 have been deposited in the European Nucleotide Archive, Accession nos. ERS2037781‐ERS2037801.

## AUTHOR CONTRIBUTIONS

P.C.S.D., B.S. and D.J.C. conceived and designed the study. K.A.K. and B.S. collected and prepared the samples. P.C.S.D. conducted the genome sequencing, bioinformatic S.N.P. calling and nucleotide data deposition. P.C.S.D., C.W.D. and D.J.C. performed data analysis and interpretation. P.C.S.D. and D.J.C. wrote the manuscript, with input from all authors.
