# Supplementary material for: Genome‐wide mosaicism in divergence between zoonotic malaria parasite subpopulations with separate sympatric transmission cycles
Source: Mol Ecol. 2018 Feb 13;27(4):860–70. doi: 10.1111/mec.14477 (PMC5918592; doi:10.1111/mec.14477)
Supplement: Supplementary file 1 [file MEC-27-860-s001.pdf]

Supporting Information

Genome-wide mosaicism in divergence between zoonotic malaria parasite subpopulations with separate sympatric transmission cycles

Paul C. S. Divis[1,2], Craig W. Duffy[2], Khamisah A. Kadir[1], Balbir Singh[1], David J. Conway[1,2]

[1] Malaria Research Centre, Faculty of Medicine and Health Sciences, Universiti Malaysia Sarawak, 94300 Kota Samarahan, Sarawak, Malaysia,
[2] Pathogen Molecular Biology Department, London School of Hygiene and Tropical Medicine, Keppel St, London WC1E 7HT, United Kingdom.

Supporting Information for this paper consists of four figures (**Figures S1 – S4**) and four tables (**Tables S1 – S4**).

Fig. S1. SNP variation in whole genome sequences of *P. knowlesi* shows three major clusters by Principal Coordinate Analysis. The 21 new samples sequenced in this study are plotted with black dots (all are within Cluster 2) while previous genome sequences are indicated with grey dots.

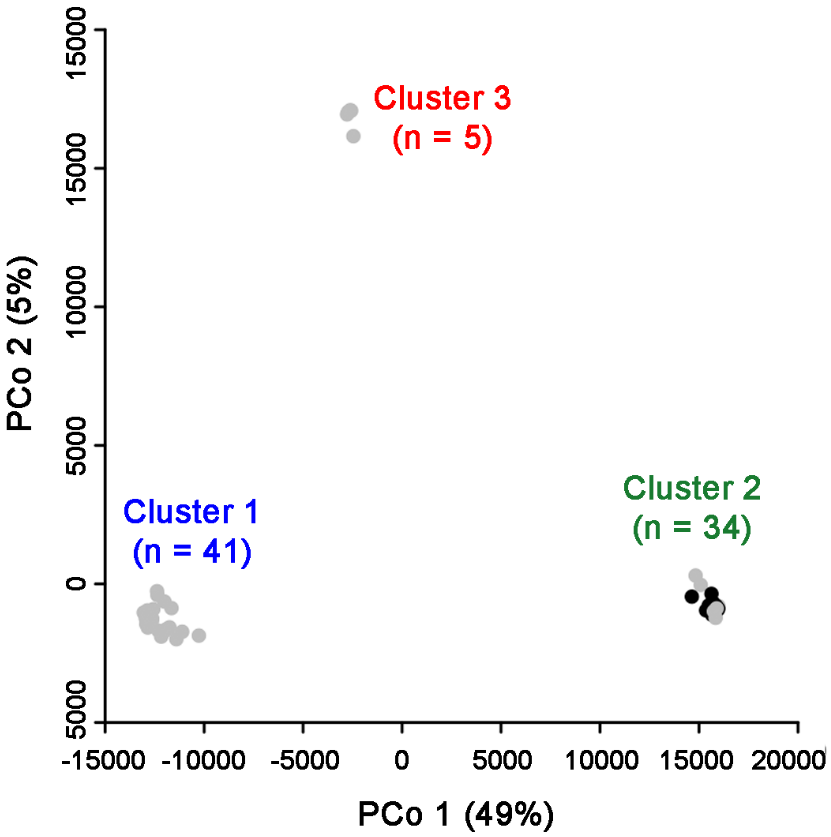

Fig. S2. Genome-wide distribution of nucleotide diversity (π) of two *P. knowlesi* subpopulation clusters in Malaysian Borneo. (A) Cluster 1 subpopulation, and (B) Cluster 2 subpopulation. Diversity was calculated in non-overlapping 10-kb windows of all 14 chromosomes. Gap between chromosomal blocks represents subtelomeric regions and not included in analysis. The mean genome-wide π for Cluster 1 subpopulation (n = 41) is 5.78×10^{-3} and for Cluster 2 subpopulation (n = 34) is 3.43×10^{-3}.

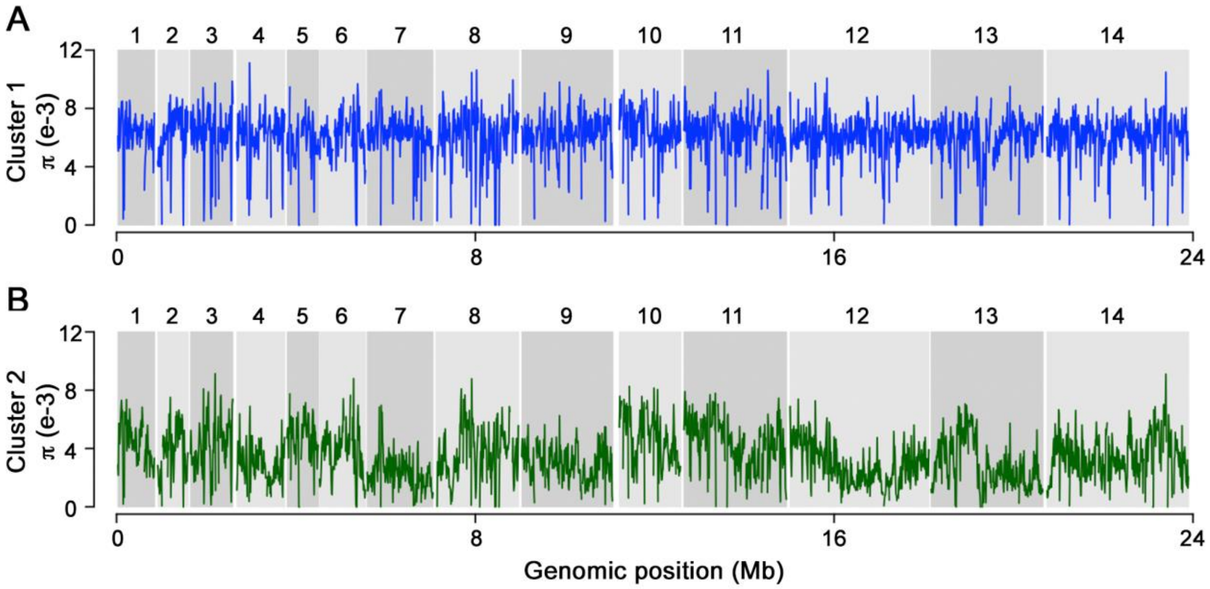

Fig. S3. Maximum Likelihood-based phylogeny inferred for mitochondrial genomes sequenced from 129 *P. knowlesi* isolates, including the samples sequenced in this study that are marked with underlined circles. Boostrap values on nodes are only shown where they are above 80% based on 1000 replicates. The scale horizontal bar indicates nucleotide substitutions per site.

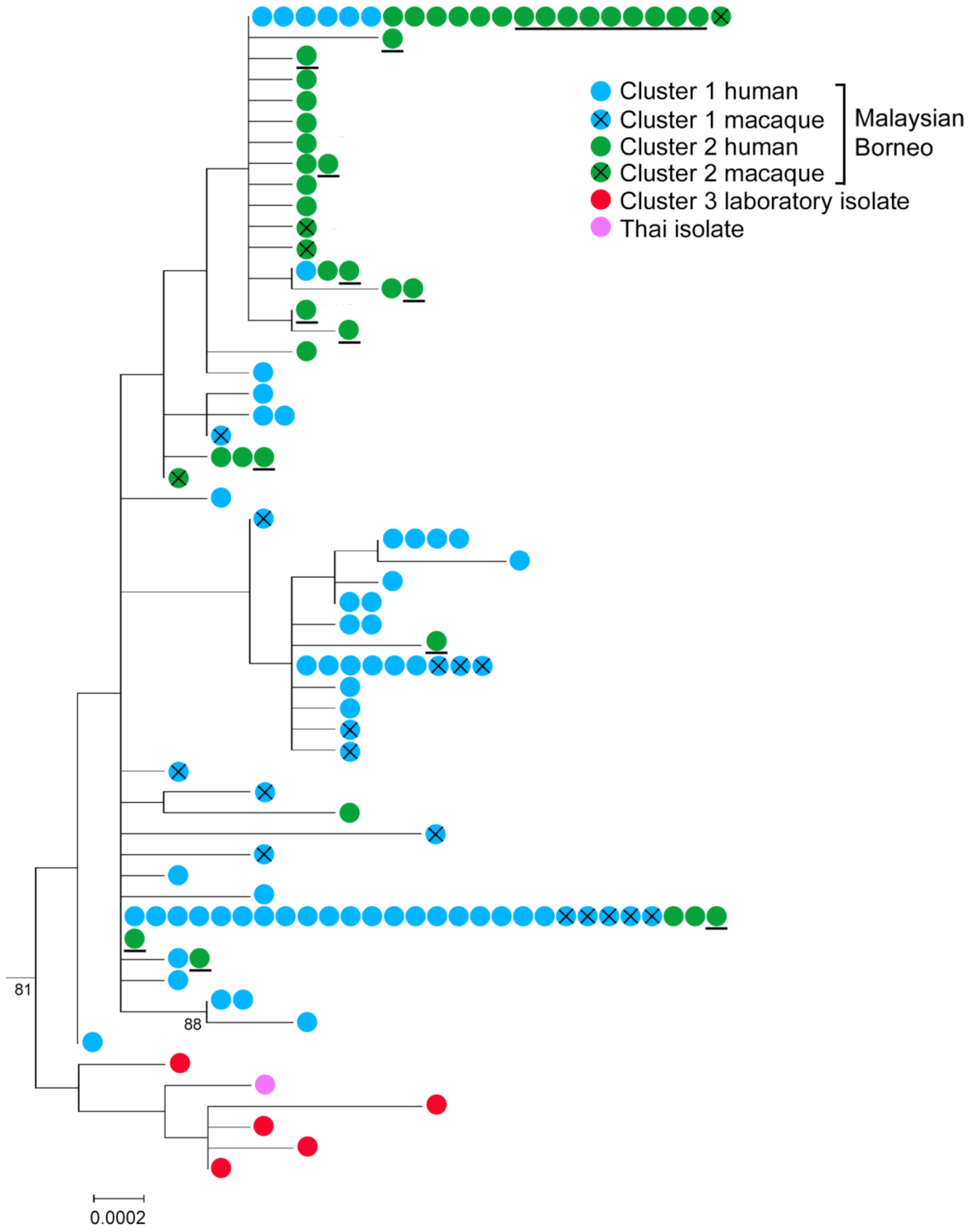

Fig. S4. Maximum likelihood phylogeny inferred by analysis of 30.6 kb of apicoplast genomes from 65 *P. knowlesi* infections, including the samples sequenced in this study that are marked with arrows. All nodes had bootstrap values above 80% based on 1000 replicates, except for those marked with black dots. The scale horizontal bar indicates nucleotide substitutions per site.

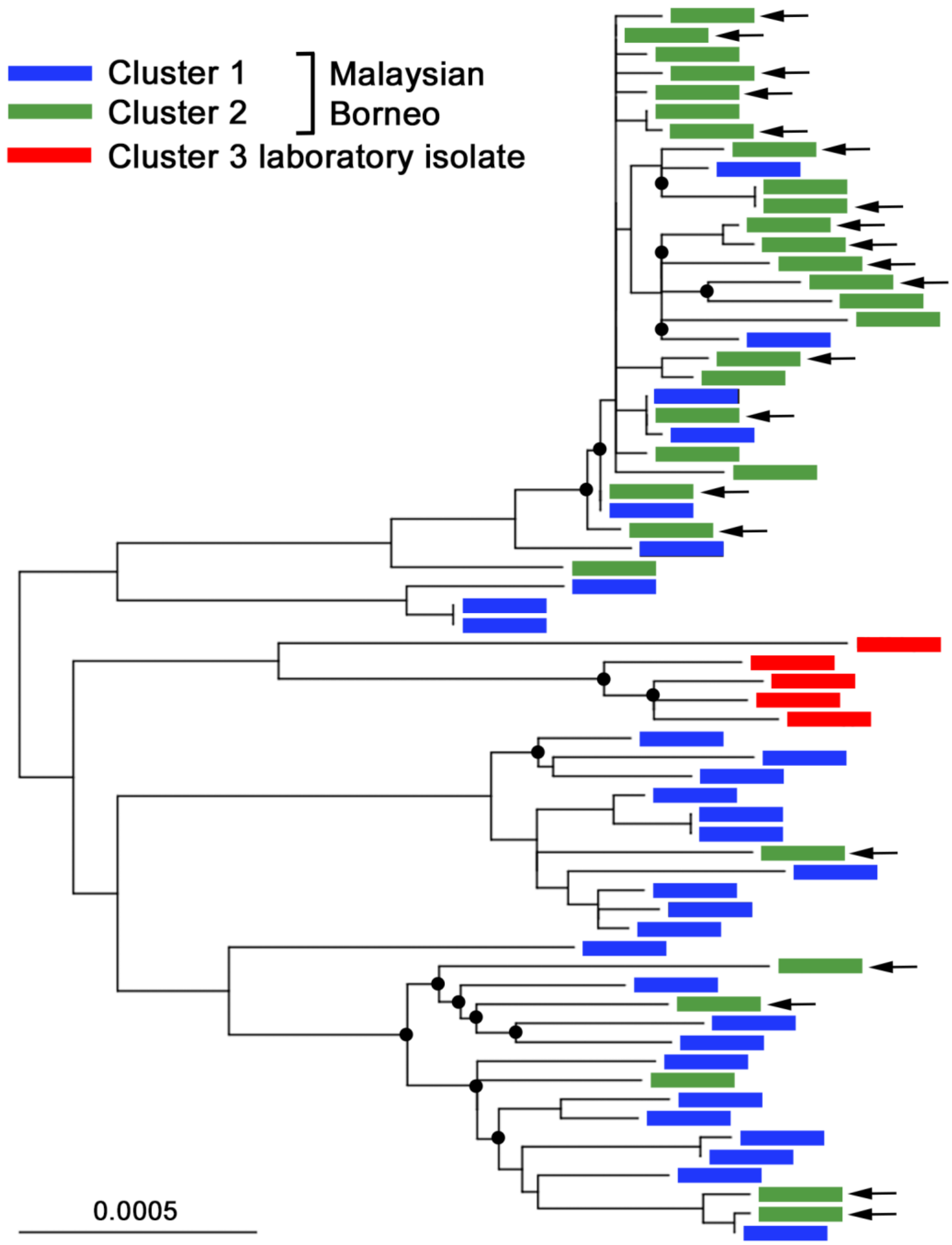

Table S1. Summary of remapping 59 previously generated short read sequences of *P. knowlesi* isolates against the version 2.0 reference genome.

Sample ID	QC-passed reads	% reads mapped	% read-pair mapped	Average depth coverage (X)
BTG026	41,313,779	96.04	84.36	146.64
BTG035	52,463,104	10.02	8.27	17.77
BTG039	45,761,870	79.59	70.55	134.72
BTG042	42,081,816	93.66	81.97	138.64
BTG044	29,325,669	97.40	86.28	106.17
BTG046	38,670,821	95.42	83.46	136.54
BTG047	32,277,653	95.26	83.92	113.71
BTG049	53,221,390	97.49	85.81	190.93
BTG050	33,519,780	94.01	80.75	115.50
BTG053	35,283,028	34.90	30.03	43.21
BTG055	21,829,275	77.64	66.57	59.30
BTG062	16,994,448	70.22	57.78	40.84
BTG063	24,682,482	62.87	54.77	54.42
BTG100	27,267,930	67.12	58.67	69.15
BTG123	25,430,647	89.20	80.91	86.82
CDK088	66,751,502	60.00	53.24	138.42
CDK206	38,122,706	35.21	30.06	46.09
KT003	42,777,884	17.76	15.00	27.47
KT004	42,025,428	92.31	81.48	146.56
KT006	60,281,143	92.74	79.15	211.51
KT012	22,442,197	95.63	85.76	81.86
KT025	56,208,103	94.16	83.79	201.71
KT026	33,338,952	83.74	73.31	105.78
KT027	52,853,787	92.96	81.42	187.58
KT029	28,872,280	62.58	54.37	68.46
KT030	28,525,157	90.61	78.79	98.13
KT031	50,367,159	90.81	81.98	173.76
KT034	23,838,990	53.04	46.11	47.20
KT040	30,655,543	97.60	86.47	113.01
KT042	28,468,946	95.15	86.27	103.16
KT048	20,775,707	77.46	68.02	60.40
KT050	19,424,602	93.49	82.97	68.46
KT055	46,135,860	90.10	84.64	156.67
KT056	48,992,338	81.75	76.99	151.34
KT057	45,715,366	92.63	85.97	159.30
KT072	45,016,497	95.70	88.65	161.03
KT073	46,334,104	95.13	88.03	164.87
KT077	26,284,061	95.29	86.43	95.54
KT081	28,061,976	95.01	86.35	101.66
KT092	26,779,552	96.35	87.61	98.40

KT094	29,726,016	97.46	88.18	110.38
KT095	26,349,673	96.90	87.82	97.27
KT100	24,359,451	97.65	88.17	90.80
KT103	22,730,860	97.59	88.17	84.31
KT107	30,273,348	94.21	85.17	108.66
KT109	26,247,181	96.40	87.09	96.43
KT114	24,462,899	88.82	81.43	83.28
KT120	20,937,609	79.02	71.65	63.15
SKS047	6,249,430	94.99	76.20	32.62
SKS048	44,612,191	97.59	86.07	165.11
SKS050A	57,817,253	98.31	86.79	215.82
SKS058	40,794,756	97.63	87.74	152.48
SKS073	6,378,060	97.36	81.60	34.48
SKS299	51,056,847	97.64	87.24	190.48
Pk_Hackeri	20,636,601	96.47	90.28	74.96
Pk_Malayan	16,464,035	87.42	86.12	55.99
Pk_MR4H	20,571,229	77.39	72.99	59.00
Pk_Nuri	23,348,408	97.30	91.52	90.08
Pk_Philippines	32,660,672	97.40	92.16	85.40

Table S2. Definition of the *P. knowlesi* subtelomeric regions excluded from analysis

**Chromosome**	**Left subtelomeric region End position (GeneID*)**	**Right subtelomeric region Start position (GeneID)**
1 PKNH_01_v2	31756 (PKNH_0100600)	851582 (PKNH_0118200)
2 PKNH_02_v2	39903 (PKNH_0200500)	717562 (PKNH_0216200)
3 PKNH_03_v2	40085 (PKNH_0300900)	984128 (PKNH_0321700)
4 PKNH_04_v2	45677 (PKNH_0400700)	1111994 (PKNH_0424800)
5 PKNH_05_v2	40025 (PKNH_0500100)	735661 (PKNH_0516400)
6 PKNH_06_v2	19789 (PKNH_0600400)	1045508 (PKNH_0623600)
7 PKNH_07_v2	9141 (PKNH_0700200)	1485805 (PKNH_0734800)
8 PKNH_08_v2	17009 (PKNH_0800400)	1874898 (PKNH_0841200)
9 PKNH_09_v2	56545 (PKNH_0900600)	2085215 (PKNH_0945900)
10 PKNH_10_v2	69857 (PKNH_1001300)	1439673 (PKNH_1032500)
11 PKNH_11_v2	29067 (PKNH_1100500)	2317969 (PKNH_1149400)
12 PKNH_12_v2	38750 (PKNH_1200400)	3129934 (PKNH_1272100)
13 PKNH_13_v2	24663 (PKNH_1300400)	2519037 (PKNH_1356400)
14 PKNH_14_v2	46265 (PKNH_1401100)	3204808 (PKNH_1472800)

*GeneIDs refer to the first and the last conserved protein-coding genes at each chromosome subtelomere boundary. These and the coordinates refer to version 2.0 of the *P. knowlesi* H strain genome sequence.

Table S3. Summary of mapping the short read sequences of 21 new isolates against the *P. knowlesi* version 2.0 reference genome.

Sample ID	QC-passed reads	% reads mapped	% read-pair mapped	Mean depth coverage (X)	Sequence Accession
KT133	8,162,583	97.83	79.73	68.43	ERSXXXXXX
KT143	5,856,181	92.50	76.18	46.80	ERSXXXXXX
KT147	4,169,555	80.77	66.14	28.66	ERSXXXXXX
KT151	10,010,749	80.95	65.17	68.03	ERSXXXXXX
KT161	10,544,255	65.18	53.45	56.19	ERSXXXXXX
KT165	8,134,708	95.69	78.58	67.36	ERSXXXXXX
KT172	7,953,445	64.71	52.08	42.17	ERSXXXXXX
KT176	5,854,135	98.42	81.19	50.11	ERSXXXXXX
KT186	9,092,261	95.21	78.00	74.47	ERSXXXXXX
KT198	7,072,188	98.30	82.09	50.96	ERSXXXXXX
KT217	8,827,711	85.51	69.74	64.04	ERSXXXXXX
KT221	6,138,392	97.72	80.20	51.73	ERSXXXXXX
KT223	7,584,970	97.07	79.87	63.69	ERSXXXXXX
KT224	10,403,400	90.68	74.10	80.27	ERSXXXXXX
KT226	7,057,397	94.60	77.89	50.92	ERSXXXXXX
KT231	3,468,323	97.98	80.38	28.99	ERSXXXXXX
KT233	5,224,563	97.59	79.88	44.10	ERSXXXXXX
KT243	9,500,408	98.33	81.53	71.51	ERSXXXXXX
KT263	4,012,790	96.38	79.36	33.64	ERSXXXXXX
KT266	6,897,861	98.00	81.77	47.88	ERSXXXXXX
KT305	3,628,341	96.37	78.70	30.26	ERSXXXXXX

All sequence accession numbers will be made publicly available upon acceptance of the article.

Table S4. Locations and lengths of high divergence regions (HDRs) and low divergence regions (LDRs) in 14 chromosomes of *P. knowlesi*

	**High-diverged regions (HDRs)**				**Low-diverged regions (LDRs)**			
**Chr**	**HDR window**	**Start position**	**End position**	**Length (bp)**	**LDR window**	**Start position**	**End position**	**Length (bp)**
1	HDR01	749494	837835	88342	LDR01	96319	679406	583088
2	HDR02	40006	180420	140415	LDR02	186366	364472	178107
					LDR03	425017	648577	238648
3					LDR04	252738	460344	207607
					LDR05	546998	937541	390544
4	HDR03	625263	919357	294095	LDR06	234546	421831	187286
5					LDR07	40804	733264	692461
6	HDR04	21165	112965	91801	LDR08	318443	864442	546000
	HDR05	921498	1024278	102781				
7	HDR06	23701	222469	198769				
	HDR07	397684	853275	455592				
	HDR08	860949	1452166	591218				
8	HDR09	26256	190875	164620	LDR09	534591	1827275	1292685
	HDR10	260538	533984	273447				
9	HDR11	1316694	1754323	437630	LDR10	765850	945549	179700
10					LDR11	70745	338054	267310
					LDR12	481963	1003531	521569
					LDR13	1067530	1385663	318134
11	HDR12	1570780	1676811	106032	LDR14	30861	1117021	1086161
					LDR15	1326585	1507915	181331
					LDR16	1681406	2194542	513137
12	HDR13	1165069	1935432	770364	LDR17	39358	729445	690088
	HDR14	1951856	2524953	573098				
	HDR15	2590082	2849921	259840				
13	HDR16	24680	146791	122112	LDR18	147119	324956	177838
	HDR17	1053698	2514922	1461225	LDR19	515520	1017446	501927
14	HDR18	46282	200282	154001	LDR20	207562	441315	233754
	HDR19	743011	915540	172530	LDR21	1036448	1221194	266306

HDR20	3103808	3186756	82949	LDR22	1363873	1539047	175175
				LDR23	2280342	2888810	608469

HDRs are regions with contiguously high F_{ST} indices between Cluster 1 and Cluster 2 (Z scores of multiple windows containing 500 adjacent SNPs all above 0.5); LDRs are regions with contiguously low F_{ST} indices (Z scores of multiple windows all below -0.5).
